# The association between gut microbiota, cholesterol gallstones, and colorectal cancer 

**Published:** 2019

**Authors:** Sama Rezasoltani, Amir Sadeghi, Ebrahim Radinnia, Ali Naseh, Zahra Gholamrezaei, Mehdi Azizmohammad Looha, Abbas Yadegar

**Affiliations:** 1 *Basic and Molecular Epidemiology of Gastrointestinal Disorders Research Center, Research Institute for Gastroenterology and Liver Diseases, Shahid Beheshti University of Medical Sciences, Tehran, Iran *; 2 *Gastroenterology and Liver Diseases Research Center, Research Institute for Gastroenterology and Liver Diseases, Shahid Beheshti University of Medical Sciences, Tehran, Iran*; 3 *Foodborne and Waterborne Diseases Research Center, Research Institute for Gastroenterology and Liver Diseases, Shahid Beheshti University of Medical Sciences, Tehran, Iran *

**Keywords:** Gut microbiota, Cholesterol gallstones, Colorectal cancer

## Abstract

Gut microbiota play critical roles in maintaining the human health in several aspects. Bile acids (BAs) are endogenous cholesterol-derived molecules that can be modified by the gut microbiota and act as signaling molecules in the regulation of host metabolic and physiology processes. Gut microbiota release many enzymes that are capable to perform considerable modifications on BAs such as bile salt hydrolases (BSH), 7α-dehydroxylase (CYP7A), and hydroxysteroid dehydrogenase (HSDH). These enzymatic roles can change in the gut microbiota composition, cause alteration in BAs profile and metabolism and even gallstone formation. Patients with 15 years of asymptomatic gallstone have increased risk for colorectal cancer (CRC), which may be related to altered gut microbiota, changes in bile metabolism, as well as cellular and molecular effects in the proximal colon. In gallstone-associated CRC patients, the association between consensus molecular subtypes of CRC should be clarified to identify if specific pathways are related.

## Introduction

**Gut microbiota**

There are 10^14^–10^15 ^bacteria residing in the human gastrointestinal tract, and estimated to outnumber the host own cells by 10 to 1 (1). The total number of bacteria in a 70 kg "reference man" has been calculated to be 3.8·10^13^ (2). The gut microbiota consists of more than 1000 different bacterial species (3), and while they vary extensively across populations, a core microbiota consisting of a fraction of these bacterial species can be recognized in large cohorts of healthy individuals (3, 4). There is a mutual relationship between the commensal intestinal microbiota and the host. Gut microbiota help in metabolism of nutrients and micronutrients the host body cannot metabolize (5). Notably, gut microbiota help control the host physiology beyond the intestinal lumen. It can signal to host body cells and control their activity (6, 7). Indeed, microbial metabolites and products such as bile acids (BAs), short chain fatty acids, amino acids, and steroid hormones are transported from the intestinal lumen environment into bloodstream and delivered to the host cells (8). These microbial metabolites are signaling molecules, and various organs of the host express certain receptors for them (9). Such receptors control cellular pathways and are exactly under the influence of microbial signaling molecules. Hence, gut microbiota plays an important role in the host physiology, even health and disease, through regulation of microbial signaling molecules for cellular receptors (Table 1) (10). Further, gut microbiota is easily changeable through environmental factors such as food diets, social contact, as well as regular probiotic and antibiotic consumption (11). Regarding the critical role of gut microbiota as a regulator of host physiology, it might be a novel and powerful candidate for treating human diseases (12).

**Impact of gut microbiota on host physiology**

 Gut microbiota interact symbiotically with the host, influence several aspects of the host's physiology (20) and produce nutrients and micronutrients in the human body. For instance, they assist in differentiation of intestinal epithelium cells (IECs), development of immune system (21), improvement of the bioavailability of nutrients in the food, elimination of the invading pathogenic bacteria (22), the gut barrier function (23), control of the host behavior (24, 25), and bone homeostasis (26). On the other hand, gut microbiota can contribute as a key regulator to host metabolic dysfunction beyond IECs (27). Further, there is a strong relationship between microbial agents and hormone production and release. Indeed, enteroendocrine cells produce and secrete a number of hormones including cholecystokinin (CCK), peptide tyrosine-tyrosine (PYY), glucagon-like peptide-1 (GLP-1), gastric inhibitory polypeptide (GIP), and serotonin (5-HT), which have regulatory roles in metabolic processes including insulin sensitivity, glucose tolerance, and fat storage. Release of these hormones can be regulated by the presence of gut microbiota and their metabolites (28).

**Bile acids**

BAs are synthesized from cholesterol in the liver and stored in the gallbladder to be secreted in the duodenal lumen upon food intake in order to facilitate fat digestion (29). BAs act on nuclear receptors, such as farnesoid X receptor, which are involved in the metabolism of triglyceride, sterol, and carbohydrate. BAs are critical signaling molecules in the host, as they interact either locally or systemically with specific cellular receptors, especially TGR5 and farnesoid X receptor (5). These functions of signaling influence systemic lipid, cholesterol, and energy metabolism, as well as intestinal electrolyte balance and immune homeostasis (30). Synthesis of BAs is under negative feedback control through activation of the nuclear receptor farnesoid X in the ileum and liver. BAs act as detergents with a critical role in solubilizing fat soluble vitamins and dietary lipids to help their absorption in the small intestine (31). Also, BAs inhibit bacterial growth or bacterial infections in the small intestine (32). In the lumen environment, microbial bile salt hydrolases (BSHs) deconjugate BAs and cause bile resistance to the gut microbiota (33). Deconjugated BAs can be absorbed more easily across the IECs and are more hydrophobic than conjugated ones (34). Then, they can be modified to secondary BAs by enzymatic activities of the intestinal bacteria. These enzymatic activities result in the modification of the primary BAs [cholic acid (CA) and chenodeoxycholic acid (CDCA)] to the secondary BAs [deoxycholic acid (DCA) and lithocholic acid (LCA)]. Overall, given the important role of microbial metabolites and function in BAs regulation and modification, commensal bacteria may be a key regulator for enterohepatic recycling of BAs, and creating resistance to *Clostridium difficile *infections (35).

**Table 1:** Gut microbiota alterations associated with different types of diseases

Bacterial agents/ Diseases	Alteration in gut microbiota composition	References
Obesity	Certain gut bacteria alterations are associated with severe obesity	(13-15)
Firmicutes/Bacteroidetes ratio↓ Bacteroidetes↓ *Lactobacillus*↑ *Methanobrevibacter smithii*↓		
Anorexia	Methanobrevibacter smithii significantly increase in lean individuals	(14, 16)
Methanobrevibacter smithii↑		
Crohn’s Disease	Changes in *Bacteroides* spp. quantity in Crohn’s disease patients are observed compared to healthy individuals	(17)
*Bacteroides ovatus*↑ *Bacteroides vulgatus*↑ *Bacteroides uniformis*↓		
Celiac disease	Higher bacterial diversity in Celiac disease patients versus control group are observed	(18)
*Bacteroides vulgatus*↑ *Escherichia coli* ↓ *Clostridium coccoides*↓		
Type 2 Diabetes	shifts in gut microbiota are associated with disease	(19)
Bacteroidetes/Firmicutes ratio ↑ *Bacteroides*- *Prevotella *↑ *Clostridium coccoides*- *Eubacterium rectale* ↓ Firmicutes ↓ Clostridia↓		

**Figure 1:**
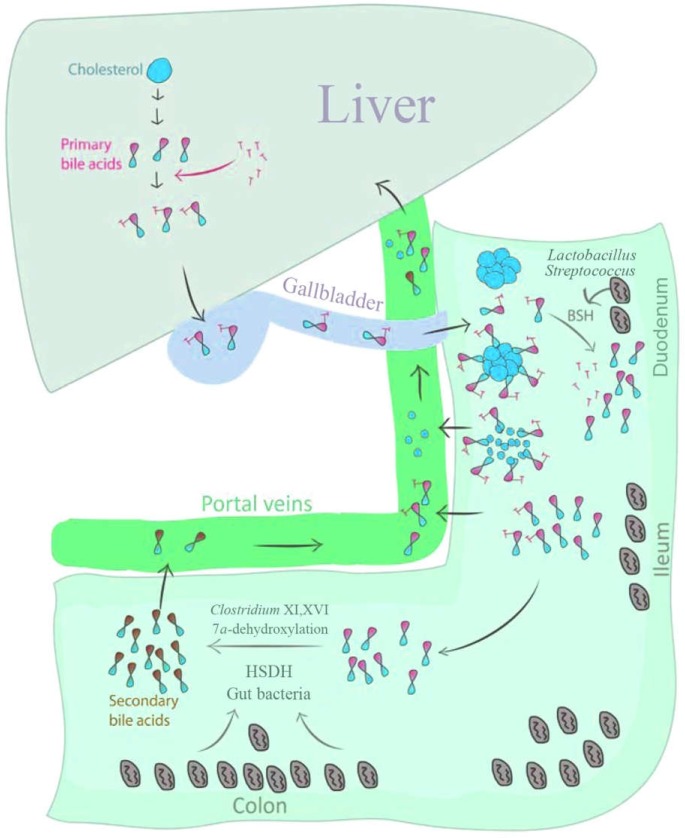
The enterohepatic circulation, BAs physiology and microbial BAs modification in the gut

**Intestinal crosstalk between BAs and microbiota**

Today, there is an increasing interest in the microbiota with respect to diseases of the gastroenterological tract including the liver and biliary system (36, 37). Potential mechanisms in the gut–liver axis terminate to gallbladder disease and carcinogenesis in different types of organ (38, 39). Gut microbiota contains many enzymes that are capable to perform modifications of BAs (40, 41). As mentioned above, the most important microbial modification of BAs in the gut is deconjugation by BSH, which is the first step of microbial modification of BAs (40) plentifully expressed by all bacterial phyla in the gut (41). Once entered into the duodenum, BAs come into contact with bacterial species with BSH activity such as *Streptococcus* and *Lactobacillus.* It is unknown why gut microbiota express BSH; it may be because deconjugation reduces BAs antimicrobial properties and toxicity to the microbiota and makes it available for subsequent modifications by the gut microbiota. On the other hand, microbial 7α-dehydroxylase enzyme is required for the production of secondary BAs deoxycholic (DCA) and lithocholic (LCA) acids. Unlike BSH, 7α-dehydroxylase enzyme has only been found in a few *Clostridium* species including *Clostridum *cluster XI and XVI which are low abundant taxa in the gut (42). Above all, hydroxysteroid dehydrogenase (HSDH) enzyme induces BAs biotransformation and is abundantly produced by gut microbiota. It catalyzes the oxidation of BAs to form oxo-BAs intermediates (42, 43). The main function of HSDH is still unrecognized, but it is speculated to generate bacterial energy through producing and reducing equivalents for cellular biosynthetic reactions (Figure 1) (40, 44). Based on these gut microbial enzymatic roles, it is thought that altered gut microbiota may be a cause and an effect of altered BAs profile and even gallstone formation. These gut microbiota alterations may further affect the intestinal epithelium cells, with progressive cellular and molecular changes in carcinogenesis (45). 

On the other hand, BAs can modulate gut microbial composition directly and indirectly through activation of innate immune genes in the small intestine. Thus, altered BAs profile may result in altered signaling via BAs receptors and affect host metabolism while also causing altered gut microbiota composition (40, 41).

**Gallstone disease and cancer risk**

In the adult population of Western countries, 10-15% develop gallstones. Cholecystectomy is a common treatment for symptomatic gallstones and other gallbladder conditions (46). As mentioned earlier, according to gut microbial enzymatic roles, it is thought that altered gut microbiota composition may be a cause and an effect of altered BAs profile and even gallstone formation (45)**. **Patients with asymptomatic gallstones at baseline had an increased risk for cancer at all gastrointestinal sites in particular for right-sided colorectal cancers (CRC). The risk of CRC increased significantly after 15 years with asymptomatic gallstone. It has been known that, cancer in certain organ sites could be explained by altered metabolic or endocrine pathways; thus, the clearest mechanism between gallstone disease and CRC may be related to altered gut microbiota, changes in bile metabolism, as well as cellular and molecular effects in the proximal colon (45, 47). Hence, gallstones contribute to increased risk for invasive CRC. Indeed, detection of certain CRC-associated bacteria raises the detection accuracy of screened CRC cases (48-50), which may be of particular importance for those with detected with silent gallstones (45). Asymptomatic gallstones may demonstrate a future CRC risk and underlying mechanism which needs to be further identified. Mechanistic links between gallstone pathology and neoplasia demand to be better understood. In particular, it should be examined how changes in bile metabolism may have local and systemic effects on human cells through molecular pathways. Overall, healthy gut microbiota can help our bodies to maintain a healthy digestion system. 

## Conclusion

Gut microbiota contains several enzymes that are capable to provide critical modifications of BAs. Based on these critical enzymatic roles, it is thought that altered gut microbiota may be a cause and an effect of altered BAs profile and even gallstone formation. Further, the association between gallstone disease and CRC may be related to altered gut microbiota, changes in bile metabolism, as well as cellular and molecular effects in the gut. Indeed, with good flora, gut inflammation will probably occur less frequently and digestive processes will run more smoothly. All of these lead to less gallstone formation. Also, changes in BAs excretion and profile are important for the human health and disease. In patients with gallstone-related CRC, any links with the consensus molecular subtypes should be explored to identify if specific pathways are associated. 

## Conflict of interests

The authors declare that they have no conflict of interest.
